# Comprehensive chemical profiling and human health risk assessment of potentially toxic heavy metals in some traditional herbal concoctions from Botswana

**DOI:** 10.3389/fphar.2025.1652817

**Published:** 2025-09-19

**Authors:** Ontlametse H. E. Phiri, Lucia M. Lepodise, Tshepo Pheko-Ofitlhile

**Affiliations:** ^1^ Department of Forensic and Chemical Sciences, Botswana International University of Science and Technology, Palapye, Botswana; ^2^ Department of Physics and Astronomy, Botswana International University of Science and Technology, Palapye, Botswana

**Keywords:** FT-IR, GC-MS, herbal concoctions, ICP-OES, risk assessment

## Abstract

Traditional herbal mixtures are still used extensively in Botswana because of their claimed therapeutic benefits, although their chemistry and safety are undocumented. Therefore, this study aims to conduct the analysis and human health risk assessment of some herbal concoctions in Botswana. The spectra of the herbal concoction samples sourced from three different street vendors were recorded at room temperature using the Fourier Transform Infrared (FT-IR) technique. The three samples exhibited comparatively similar spectral profiles, indicating that they may contain chemical compounds vibrating at similar energies. FT-IR analysis revealed the presence of characteristic functional groups which includes phenolics, alcohols, alkene, alkanes and aromatic groups some of which were identified through gas chromatography–mass spectrometry (GC-MS) analysis. GC-MS analysis of all the hexane extracts identified octane and 3-methylheptane as the major constituents. Hexadecane, tetradecane and 5-Aminovaleric acid were major compounds identified in ethyl acetate extracts. Inductively Coupled Plasma Optical Emission Spectrometer (ICP-OES) results revealed that the heavy metal concentrations of herbal concoctions that ranged between 1.00 × 10^−4^ mg/kg and 2.43 × 10^1^ mg/kg. The concentrations of all the heavy metals were below the acceptable limits set by World Health Organization (WHO). Trace metal concentrations of Mg, Ca, K and Na in the samples ranged from 1.31 × 10^−1^ to 2.79 × 10^1^ mg/kg, 4.34 × 10^−1^ to 2.36 × 10^2^ mg/kg, 1.13 to 2.26 × 10^1^ mg/kg and 3.08 × 10^−1^ to 5.07 × 10^1^ mg/kg respectively. The human health risk analysis showed that there was no potential health risk associated with the consumption of the herbal concoctions and As concentration levels in sample A requires close scrutiny. These findings provide valuable information on the chemical composition, metal content and health risk assessment information of the herbal concoctions by informing safe usage and contributing to evidence based ethno-pharmacological research. The study provides an insight on the properties of bioactive compounds present in the herbal concoctions and emphasizes the necessity of continuous quality monitoring and chemical validation of traditional herbal mixtures to inform regulatory frameworks and public health policy.

## 1 Introduction

Herbal concoctions are remedies made from the combination of different plant species or plant components for treatment of a wide range of illnesses. These mixtures can be made from both fresh and dried plant materials. The plant materials can either be boiled or macerated in water for several days (Ndhlala and Van Staden, 2012; Matotoka and Masoko, 2018). They often contain minerals and trace metals, among other highly active pharmaceutical ingredients (Fabricant and Farnsworth, 2001). Trace elements are involved in a wide range of metabolic processes, ranging from signal transduction to gene regulation, energy metabolism, hormone sensing, and primary and secondary metabolism to cell defense (Vatansever et al., 2017). Flavonoids, tannins, alkaloids and phenolic compounds are some of the phytochemicals present in these herbal mixtures that serve as active pharmacological compounds. About 70%–80% of the population globally, still depend on nonconventional medications which are mostly sourced from plants (Sahoo et al., 2010; World Health Organization, 2002). Low and middle income countries have 20%–80% of dependence on herbal medicine for healthcare needs and this includes countries like South Africa (Singh et al., 2004), Trinidad (Merritt-Charles et al., 2003; Clement et al., 2007), Nigeria (Danesi and Adetunji, 1994; Amira and Okubadejo, 2007) and the Caribbean (Michie, 1992; Gardner et al., 2000).

Toxicity due to heavy metals, adulteration, microbial and pesticide contamination, and the overall processing of plant extracts are some of the challenges associated with using herbal medicine (Ernst, 2002; Saper et al., 2004; Adeleye et al., 2005; Jung et al., 2006; Tilburt and Kaptchuk, 2008; Ndhlala et al., 2013; Ekor, 2014). Additionally, pesticides usage and other agrochemicals, plants growing alongside busy roads, former landfills, and areas close to mining sites have all been linked to elevated amounts of heavy metals in medicinal plants (Bempah and Ewusi, 2016; Street, 2012). Medicinal plants near the farming lands, factories and mining areas in Turkey were found to contain elevated concentrations of heavy metals like lead (Pb), arsenic (As), chromium (Cr), cadmium (Cd), zinc (Zn) and nickel (Ni) amongst others (Ozyigit et al., 2022). There is a common misconception of natural herbs and medicinal plants being harmless and safe; however there are documented reports on the toxicity and negative side effects associated with the usage of herbal plants and their formulations around the globe (Ernst, 2002; Okareh et al., 2018).

A study on herbal concoctions at Ga Maja, Limpopo province in South Africa revealed the presence of trace and heavy metals, and they also exhibited antioxidant and antimicrobial activities (Matotoka and Masoko, 2017). In Iran, Pb and Cd concentrations were above allowable concentrations levels set by WHO in the studied herbal mixtures (Mousavi et al., 2014). The Ni concentrations in some branded herbal mixtures were in the range of 0.2–56.3 mg/kg (Saeed et al., 2010). High Ni concentrations of 0.48–76.97 mg/kg were recorded in some herbal products at Karachi city in Pakistan (Hina et al., 2011). Ten (10) different liquid herbal formulations sold in Nigeria have Cr and Zn concentrations ranges from 0.00150 to 0.0750 mg/kg and 0.329–1.23 mg/kg respectively (Izah et al., 2022). Anwar et al. (2024) reported high hazard quotient (HQ) values for As in all the studied traditional herbal medicines at Khyber Pakhtunkhwa in Pakistan.

Some herbal tea products and food stuffs are reported to be unsafe for consumption due to heavy metal contamination (Bortey-Sam et al., 2015; Nkansah et al., 2016). Cancer, liver, kidney toxicities, genetic mutations and central nervous system diseases are some of the documented health issues linked to heavy metals (Shaban et al., 2016). The potential health risks associated with exposure to As are diabetes mellitus, high blood pressure, cancer or skins lesions (Yunus et al., 2011; Chakraborti et al., 2016) while the health complications which include nephrotoxicity, endometrial cancer and cardiovascular health issues are associated with Cd exposure (McElroy et al.,2017; Garner and Levallois, 2016). Exposure to elevated quantities of hazardous metals found in herbal concoctions, may pose major health hazards in the long term consumption of the herbal concoctions, hence a need to access the chemical and elemental composition of the herbal concoctions. Previous studies on human health risk assessment on plants and herbal formulations are geographically limited to countries in North Africa and a few in southern Africa using advanced chromatographic and spectroscopic techniques (Al-Keriawy et al., 2023; Durodola et al., 2019; Sekwati-Monang et al., 2018). While some ethnobotanical, ethnopharmacological survey, antioxidant and antimicrobial studies has been conducted in different medicinal plants and herbs (Motlhanka and Ntlhoiwa, 2013; Richard et al., 2023) from Botswana, there is a knowledge gap on chemical profiling of the traditional herbal concoctions sold and used by rural communities especially at Dibete. Despite limited studies on the traditional herbal medicine from Botswana (Setshogo and Mbereki, 2011), local communities at Dibete, Botswana often rely on traditional herbal medicine even with lack of information on their chemical composition, safety and health risk associated with the consumption of the herbal concoctions. This work presents a foundation for pharmacological and safety profile investigations of traditional herbal concoctions in Botswana by bridging traditional medicinal knowledge, scientific techniques and documentation. Therefore, this study aims to explore the chemical profile and metal composition (trace and heavy metals) as well as human health risk associated with the consumption of the traditional herbal concoctions from Botswana.

## 2 Materials and methods

### 2.1 Sample collection and preparation

Traditional herbal concoctions (Sample A, Sample B and Sample C) were bought from three different local street vendors around Dibete area, Central District, Botswana (23.7475° S, 26.4681° E). The studied herbal concoctions are widely sold and consumed at Debete, claimed to treat gastro-internal issues, urinary tract infections and have not been previously subjected to chemical profiling in scientific literature. The three independent street vendors prepared the herbal concoctions (Sample A, Sample B and Sample C) by boiling a mixture of different plant species in water using large pots (Table 1). The cooled decoctions were sold in recycled 2 L bottles. After collection, the freshly prepared herbal concoctions were all put in a non-light penetrative cooler box, maintained at 4 °C and transported to the laboratory where analysis was carried out. Each herbal concoction sample was labelled and stored at temperatures 4 °C ± 2 °C until future use. Prior to any analysis, the labelled herbal concoctions (Sample A, Sample B and Sample C) were subjected to gravity filtration for removal of any insoluble plant residues.

**TABLE 1:** Plant species used in the preparation of herbal concoctions.

Species name	Local name (setswana name)	Sample A	Sample B	Sample C
*Elephantorrhiza goetzei*	Motsitsane	x	-	-
*Indigofera flavican* Bak.	Tshikadithata	x	x	x
*Dicoma Anomala*	Tshipisemagale	x	-	x
*Eulophia hereroensis*	Magorometsa	x	-	x
*Cissus quadrangularis*	Leme-le-monate	x	-	-
*Ximenia americana L.*	Moretologa	x	-	-
*Loranthaceae* spp.	Palamela	x	-	-
*Asparagus africanus (Lam.*) Oberm	Mhalatsamaru	x	-	x
*Melia azedarach*	Morolwana	x	-	-
*Urginea sanguinea* Schinz	Sekaname	x	-	-
*Harpagophytum procumbers* (Burch.) DC	Sengaparile	x	x	-
*Protoaspagus spp*	Radipolwana	-	x	x
*Tragia brevipes*	Motsatsana	-	x	x
*-*	Mmatlakgomo	-	x	-
*Hypoxis hemerocallidea* Fisch. Mey & Ave-Lall.	Tshuku-ya-poo	-	x	-
*Artemisia afra*	Kgomoyamaburu	-	-	x
*Dichrostachys cinerea* (L.) Wight & Arn. sensu lato	Moselesele	-	-	x

Key: Present: (x) Absent (-).

Table 1 presents the different plant species that each vendor claimed to have used in the preparation of the herbal concoctions.

### 2.2 Chemicals and solvents

Ethyl acetate and *n*-hexane were purchased from Rochelle chemicals, South Africa and hydrochloric acid (32%) from Minema chemicals (Pty) Ltd., South Africa. Ca, Mg, Mn, Na, As, Cd, Cr, Pb, Zn, Cu and K standards were purchased from Sigma Aldrich, USA. All the chemicals used were of pure analytical grade.

### 2.3 Analytical methods

#### 2.3.1 FT-IR analysis

A Vertex 70v Bruker spectrometer equipped with the Bruker Attenuated Total Reflection accessory was used to record the infrared spectra of the samples. The detector used was the deuterated triglycine sulfate (DTGS) and the source of radiation was the tungsten lamp. A broadband beam splitter was employed. The average number of scans was set at 512 while the resolution at all frequencies was set at 4 cm^−1^. All the samples were subjected to gravity filtration and a drop of the filtrate was placed in a diamond crystal and measurements were done under vacuum.

#### 2.3.2 Gas-chromatography-mass spectrometry (GC-MS) analysis

The literature protocol by Phiri et al. (2021), was used for GC-MS analysis of the herbal concoction samples with some modifications. The GC-MS analysis of the hexane and ethyl acetate extracts of the three herbal concoction samples (A, B and C) was determined using Agilent 7890B GC, coupled to an Agilent 5977A mass spectrometer detector. The HP-5 MS capillary column (Hewlett-Packard, CA, United States) which has a stationary phase of 95% dimethyl silicone layered with 5% phenyl-methyl silicone of phase thickness of 0.25 mm was employed. The column dimensions were 30 m × 320 μm × 0.25 mm with 0.25 thickness. Helium gas was used as a carrier gas. The flow rate, pressure and average velocity employed were 0.5 mL/min, 3.1561 psi and 10.769 cm/s respectively. The mass spectrometer detector and injector temperature were set at 250 °C. The initial temperature of the oven was set at 250 °C and was held for 5 min, then increased to 300 °C at a rate of 5 °C/min. The electron impact at 70 eV was utilized as the ionization mode of the detector. The selected mass range was 40–300 m/z. The identification of volatile chemical compounds was performed based on GC retention times, on an HP-5 MS capillary column (Hewlett-Packard, CA, United States) and by matching the MS data obtained with the National Institute Standard and Technology (NIST 2012 version) database. The spectra that displayed above 95% match was selected for the identification of chemical constituents. The relative percentage composition of the compounds was computed based on the peak areas (Akwu et al., 2019). GC-MS analysis was primarily employed for qualitative profiling of the bioactive compounds present in the studied solvent extracts. Therefore, MLDs were not calculated as the calibration curves were not employed for quantification.

### 2.4 Elemental analysis

#### 2.4.1 Sample digestion

The crude samples A, B and C (1 g) were each mixed with 10 mL of HCl (6 M) in a beaker, then heated at 50 °C using a hot plate for 24 h. Whatman No. 1 (90 mm diameter filter paper of pore size ∼11 µm) was used for gravity filtration of the digested samples. The samples were then diluted with 50 mL deionized water and then filtered three times using a 10 mL polypropylene Whatman syringe and filter of pore size 0.45 µm. The filtered test samples were then analysed for trace and heavy metals (Albakaa et al., 2021).

#### 2.4.2 Standard solution preparation

The standard solutions of calcium (Ca), aluminum (Al), Mg, manganese (Mn), Cr, Na, As, Cd, Pb, Zn, Cu and K were prepared from 1,000 mg/L stock solutions (Sigma Aldrich, United States). The stock solutions were diluted to make standards solutions in a 50 mL volumetric flask and filled to the volumetric mark using distilled water. The prepared standards concentrations for calibration curves of Ca, Mg, Mn, Na, As, Cd, Cr, Pb, Zn, Cu and K were in the range of 0.02 mg/L to 10 mg/L.

#### 2.4.3 ICP-OES parameters

Thermo iCAP 7400 ICP-OES-Duo (Thermo Fisher, Germany) was employed for metal analysis. iTEVA operating software for iCAP 7400 series was used to control all functions of the instrument. For trace and heavy metal elements determination, axial mode and radial mode were selected respectively. The elements analyzed were Ca, K, Mg, Na, Mn, As, Cd, Cr, Cu, Pb and Zn with selected emission wavelengths (nm); 422.6, 766.4, 202.5, 589.5, 257.6, 189.0, 228.8, 283.5, 324.7, 220.3, and 213.8 respectively. The RF power, auxiliary gas flow and argon nebulizer gas flow were set at 1150 W, 0.5 L/min, 0.70 L/min respectively. Pump stabilization time, flush time, analysis pump rate, and flush pump rate were all set at 5 s, 30 s, 50 rpm, and 100 rpm, respectively. To measure the analytical signal, 3 replicates and 2-point background correction were done.

##### 2.4.3.1 Limit of detection

Equation 1 was used to evaluate the instrument’s limit of detection (LOD), by dividing three times standard deviation (SD) of blank (b) absorbance triplicates by the slope (m) calibration curve. The instrument LOD values (mg/kg) for Ca, Mg, Mn, Na, As, Cr, Pb, Zn, Cd, Cu and K are 1.62 × 10^−3^, 2.25 × 10^−3^, 4.37 × 10^−4^, 1.19 × 10^−2^, 8.16 × 10^−4^, 1.28 × 10^−3^, 2.90 × 10^−3^, 1.06 × 10^−3^, 3.77 × 10^−5^ 2.24 × 10^−3^ and 6.55 × 10^−3^ respectively.

$$\text{LOD}=\frac{3\;*\;SDb}{m}$$ (1)

#### 2.4.4 Human health risk assessment of heavy metals in herbal concoctions

The estimated daily intake (EDI), non-carcinogenic risk assessment (target hazard quotient and hazard index) and cancer risk (CR) were evaluated to determine the potential health impacts of the consumption of the studied herbal concoctions.

##### 2.4.4.1 Estimated daily intake of heavy metals in herbal concoctions

Equation 2 was used to evaluate the estimated daily intake (EDI) of the studied heavy metals (As, Cd, Cr, Cu, Pb, Zn and Mn) in the samples (Durodola et al., 2019).

$$\text{EDI}=\frac{C\;*\;IR\;*\;EF\;*\;ED}{BW\;*\;AT}$$ (2)

Where, C is elemental concentrations of the herbal concoctions (mg kg^−1^), IR is ingestion rate (kg person^−1^ day^−1^) (0.008 kg person^−1^ day^−1^ for adults), EF is exposure frequency (365 days year^−1^), ED is exposure duration (70 years for adults), BW is average body weight (kg), (70 kg) and AT is average exposure time for non-carcinogens (365 days year^−1^ × ED). The international reference dose values for the heavy metals (RfD) (mg kg^−1^ day^−1^) used were: 0.02, 0.14, 0.001, 0.003, 0.001, 0.004 and 0.3 for Cr, Mn, Cu, As, Cd, Pb and Zn respectively (FAO/WHO Codex Alimentarious Commission, 2013; USEPA, 2015). The assumptions for the health risk assessment calculations where the ingested dose is equal to the absorbed pollutant dose (USEPA, 1989) and the average body weight (BW) of adults consuming the herbal concoctions was assumed to be 70 kg.

##### 2.4.4.2 Target hazard quotient (THQ)

Target hazard quotient (THQ) is a ratio of the exposure dose (EDI) to the Reference dose (RfD). The standard assumptions from the integrated USEPA risk analysis were used to calculate the dose estimations (USEPA, 2000). The non-carcinogenic risk (Target hazard quotient) was calculated using Equation 3.

$$\text{THQ}=\frac{EDI}{RfD}$$ (3)

##### 2.4.4.3 Hazard index (HI)

The hazard index (THQ) values were expressed as summation of the individual metal target hazard quotient (THQ) values. The hazard index was calculated using Equation 4 (Chien et al., 2002; Ullah et al., 2017)

$$\text{HI}=\text{THQ }\left(\text{Toxicant }1\right)+\text{THQ }\left(\text{Toxicant }2\right)+\text{THQ }\left(\text{Toxicant }3\right)+\ldots ..\text{THQ }\left(\text{Toxicant }n\right)$$ (4)

##### 2.4.4.4 Cancer risk estimation

The cancer risk over a lifetime of exposure of cancer metals was calculated using Equation 5.

CR=EDI * CSF (5)

Where EDI is the estimated daily intake of heavy metals and CSF is the cancer slope factor of 0.5, 1.5, 8.5 × 10^−3^ (mg/kg/day)^−1^ for chromium (Cr), arsenic (As) and lead (Pb) respectively (USEPA, 1989; CalEPA, 2019; USEPA, 2009).

### 2.5 Statistical data analysis

Descriptive statistics were performed for evaluation of risk assessment parameters, namely, estimated daily intake (EDI), target hazard quotients (THQ), hazard index (HI) and cancer risk (CR) estimations by employing Monte Carlo simulation (10,000 iterations) to account for variability and uncertainty in metal concentrations with 50th percentile (P50) and 95th percentile (P95) performed in each parameter. One sample t-test was performed to determine whether the heavy metal concentrations in the samples significantly differs with WHO permissible elemental concentrations. The 95% Confidence intervals (CI) of means and coefficient of variation (CV %) were calculated to evaluate the uncertainty around the mean values and metal concentrations’ relative variability respectively.

## 3 Results and discussions

**FIGURE 1:**
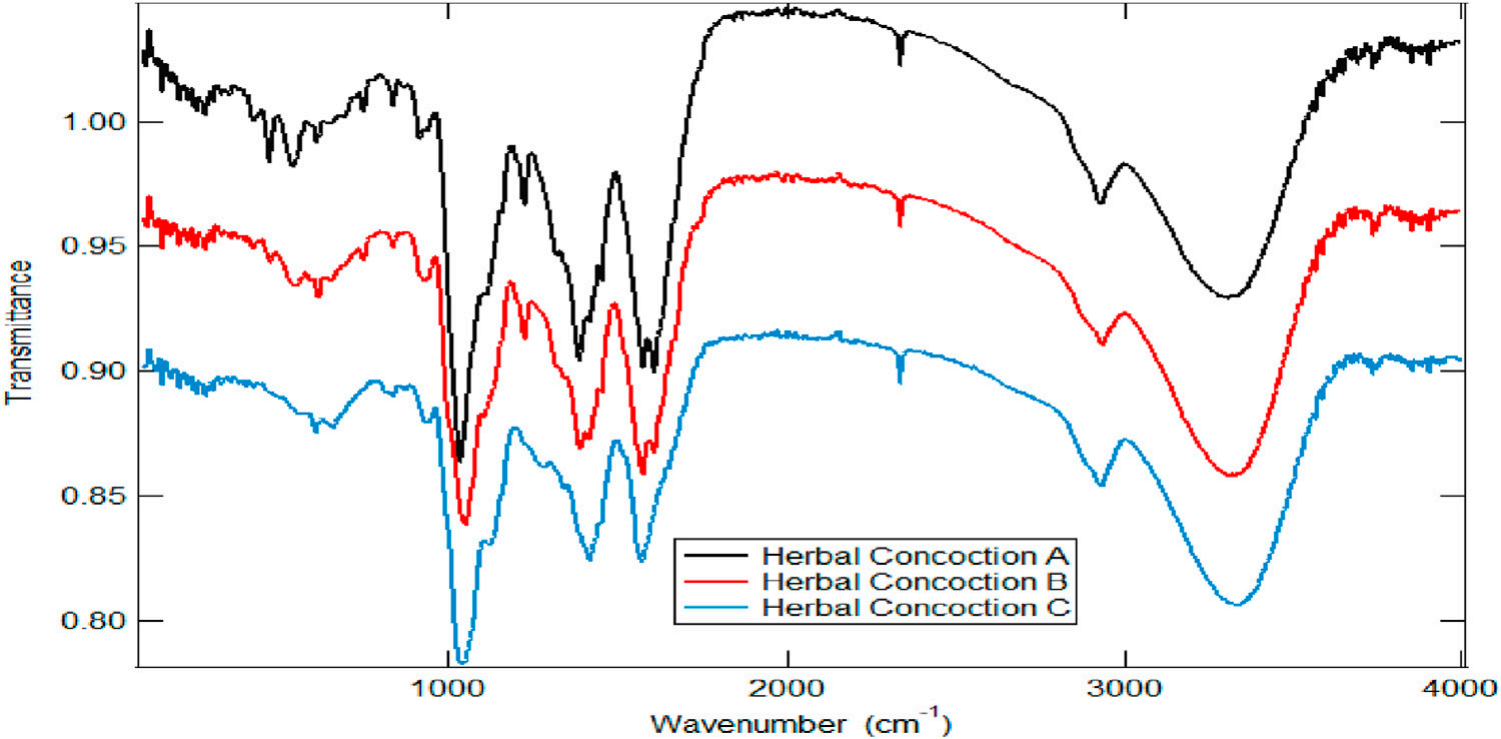
The FTIR spectra of the herbal concoctions A, B and C for different street vendors.

Figure 1 shows the infrared spectra of the crude herbal concoction samples A, B and C. FT-IR spectroscopy was employed for functional group identification in the herbal concoctions. Absorption bands at 3739.20–3313.00 cm^−1^, 3741.60–3280.80 cm^−1^ and 3736.50–3310.40 cm^−1^ for herbal concoction A, herbal concoction B and herbal concoction C respectively, suggest the presence of hydroxyl groups (Coates, 2000; Park et al., 2015; Smith, 2018). Chemical compounds with OH functional group were identified through GC-MS profiling of the samples (Tables 2, 3). The presence of wide O-H stretch across all the samples may also be attributed to water as these herbal concoctions are prepared using water. A study by Wongsa et al. (2022) on twenty (25) herbal infusions shows absorption band 3400–3200 cm^−1^ which is indicative of hydroxyl groups and H-bonded stretching which characterizes phenolic compounds. Phenolic compounds are common, widely spread in plant kingdom and present in different plant species and tissues (Balasundram et al., 2006; Zagoskina et al., 2023). The sharp peaks at 2926.30 cm^−1^, 2933.90 cm^−1^ and 2932.30 cm^−1^ of herbal concoctions A, B and C as shown in Figure 1, are indicative of CH, –CH_2_ and -CH_3_ stretch vibrations (Shurvell, 2002), which maybe suggestive of alkanes as identified in the samples through GC-MS profiling (Tables 2, 3). The asymmetrical and symmetrical aromatic C=C stretch which are hinted by absorption bands at 1578.40–1414.30 cm^−1^, 1594.60–1391.70 cm^−1^ and 1590.20–1455.90 cm^−1^ for herbal concoction A, herbal concoction B and herbal concoction C respectively, may suggest the presence of aromatic groups and unsaturated linkages (Kubovsky et al., 2020). Regions at 840–627 cm^−1^ maybe assigned to substitution patterns at Ar-H group (Vihakas, 2014). Chemical constituents with C-N, C-O and P-O bonds are associated with the fingerprint region (Coates, 2000). The two absorption peaks in the finger print region at 833 cm^−1^ and 518 cm^−1^ depicting C-H and C-C bending that is out of plane may hint the presence of 1,4– di-substituted benzene compound(s) as previously reported by Heredia-Guerrero et al. (2014). These results show that FT-IR as a quick, non-destructive spectroscopic technique can rapidly characterize plant materials through functional group analysis in real time and highlight the differences and similarities in the herbal concoctions.

**TABLE 2:** GC-MS analysis of Sample A, B and C hexane extracts.

Compound name	Chemical formula	Class	Retention time (Min)	Relative percentage abundance (%) of herbal concoctions hexane extracts		
				A	B	C
2-methylheptane	C_8_H_18_	Alkane	3.314	-	28.1	-
			3.335	26.3	-	22.5
3-methylheptane	C_8_H_18_	Alkane	3.479	-	30.4	-
			3.438	29.1	-	-
Octane	C_8_H_18_	Alkane	3.767	-	11.0	-
			3.788	10.5	-	33.2
Decane	C_10_H_22_	Alkane	4.241	-	-	0.42
4-methyl- octane	C_9_H_20_	Alkane	5.003	-	6.07	-
			5.004	5.83	-	-
*p*-xylene	C_8_H_10_	Aromatic hydrocarbon	5.230	11.4	11.5	-
			5.251	-	-	9.59
Nonane	C_9_H_20_	Alkane	5.931	-	3.39	3.31
6-methyl-octadecane	C_19_H_40_	Alkane	5.930	3.20	-	-
			4.365	-	2.58	-
			29.400	-	-	0.64
Undecane	C_11_H_24_	Alkane	12.236	-	-	0.60
Dodecane	C_12_H_26_	Alkane	15.409	-	-	0.35
2,6-dimethyl- heptadecane	C_19_H_40_	Alkane	21.096	-	-	0.39
Hexadecane	C_16_H_34_	Alkane	26.021	-	2.16	0.73
Butyl ester-6-tetradecane-sulfonic acid	C_18_H_38_O_3_S	Ester	29.421	-	-	0.64
2,4,7,14-Tetramethyl-4-vinyl-tricyclo[5.4.3.0(1,8)]tetradecan-6-ol	C_20_H_34_O	Alcohol	33.871	-	-	0.39
Cholestan-3-ol, 2-methylene-, (3β,5α)	C_28_H_48_O	Alcohol	34.490	-	-	0.41
Methyl ester-11,14-Eicosadienoic acid	C_21_H_38_O_2_	Fatty acid methyl ester	35.767	-	-	0.51
5-methyl-heneicosane	C_22_H_46_	Alkane	37.209	-	1.58	0.84
(Z)-5-Methyl-6-heneicosen-11-one	C_22_H_42_O	Ketone	39.229	-	-	1.02
Octadecamethyl- cyclononasiloxane	C_18_H_54_O_9_Si_9_	Cyclosiloxane	39.517	4.04	-	-
(Z)-9-Octadecenamide	C_18_H_35_NO	Fatty amide	40.939	5.56		-
3,4′,5,6′-tetrakis(1,1-dimethylethyl)-[1,1′-biphenyl]-2,3′-diol	C_28_H_42_O_2_	Phenol	40.980	-	-	1.01
3-ethyl-5-(2-ethylbutyl)- octadecane	C_26_H_54_	Alkane	41.413	-	-	2.60
1-Monolinoleoylglycerol trimethylsilysl ether	C_27_H_54_O_4_Si_2_	Ether	41.825	1.58	-	-
			45.348	-	-	0.86
Heptacosane	C_27_H_56_	Alkane	43.000	-	-	0.74
Oleic acid, 3-(octadecycloxy)propyl ester	C_39_H_76_O_3_	Fatty acid ester	43.535	-	-	1.20
3′,8,8′-Trimethyl-3-piperidyl-2,2′-binaphthalene-1,1′,4,4′-tetrone	C_31_H_29_N_2_O_4_	Quinonoid aromatic compound	43.844	-	-	1.12
1,1’-[1,3-propanediybis(oxy)]bis-octadecane	C_39_H_80_O_2_	Alkane	45.987	-	-	0.61
Alkane’s identified (%)				74.9	85.3	66.9
Amides identified (%)				5.56	-	-
Aromatic hydrocarbons identified (%)				11.4	11.5	11.7
Ethers identified (%)				1.58	-	2.35
Organo-silicon compounds identified (%)				4.04	-	-
Esters identified (%)				-	-	2.35
Ketones identified (%)				-	-	1.02
Percentage of identified compounds (%)				97.5	96.8	83.7

(−): not applicable.

**TABLE 3:** GC-MS analysis of Sample A, B and C ethyl acetate extracts.

Compound name	Molecular formula	Class	Retention time (Min)	Sample names		
				A	B	C
				Relative percentage abundance (%)		
Cis-1,3-dimethylcyclohexane	C_8_H_16_	Cyclic alkane	3.591	6.51	6.07	4.03
Trans-1-ethyl-3-methylcyclopentane	C_8_H_16_	Cyclic alkane	3.733	-	-	4.77
			3.753	7.47	6.58	-
Bicyclo[2.1.1]hexan-2-ol, 2-ethenyl	C_8_H_12_O	Alcohol	5.307	-	-	0.15
Benzene propionic acid, 4-tridecyl ester	C_22_H_36_O_2_	Ester	5.892	1.76	-	-
Nonane	C_9_H_20_	Alkane	5.932	-	-	1.34
O-decyl-hydroxylamine	C_10_H_23_NO	Hydroxylamine derivative	5.932	-	1.64	-
p,α-dimethyl- phenethylamine	C_10_H_15_N	Amine	7.930	-	-	0.17
Decane	C_10_H_22_	Alkane	8.959	1.76	-	-
			8.979	-	5.23	3.91
N-α,N-ω-Di-cbz-L-arginine	C_22_H_26_N_4_O_6_	Amide	10.291	-	-	0.81
Carbamothioic acid, O-isopropyl ester	C_4_H_9_NOS	Carbamate ester	10.997	-	-	0.18
3-(4-isopropylphenyl)-1,1-dimethylurea	C_12_H_18_N_2_O	Amide	11.582	-	-	0.17
Undecane	C_11_H_24_	Alkane	12.268	7.42	-	-
			12.288	-	-	5.25
			12.289	-	7.11	-
Dodecane	C_12_H_26_	Alkane	15.396	7.06	-	-
			15.416	-	6.66	-
Oxalic acid, isobutyl nonyl ester	C_15_H_28_O_4_	Ester	15.457	-	-	4.91
2-Aminononadecane	C_19_H_41_N	Amine	18.362	-	-	0.27
Cyclohexasiloxane, dodecamethyl-	C_12_H_36_O_6_Si_6_	Siloxane	19.129	-	-	0.19
5-Aminovaleric acid	C_5_H_11_NO_2_	Delta-amino acid	20.945	-	-	10.5
Tetradecane	C_14_H_30_	Alkane	21.026	14.7	14.3	-
N-methyl-1-octadecanamine	C_19_H_41_N	Amine	23.003	-	-	0.31
3-methylheneicosane	C_22_H_46_	Alkane	23.629	-	0.78	-
N-2-Hydroxyethylpiperazine-N-3-propanesulfonic acid	C_9_H_20_N_2_O_4_S	Sulfoalkylpiperazine derivative	23.669	-	-	0.82
1H-Inden-5-ol, 2,3-dihydro-3-(4-hydroxyphenyl)-1,1,3-trimethyl-	C_18_H_20_O_2_	Polycyclic aromatic alcohol	25.304	-	-	0.53
Hexadecane	C_16_H_34_	Alkane	25.990	15.2	-	-
			26.010	-	-	10.4
Nonadecane	C_19_H_40_	Alkane	26.010	-	13.9	-
2-t-Butyl-3-methyl-1-(toluene-4-sulfonyl)imidazolidin-4-one	C_15_H_22_N_2_O_3_S	Imidazolidinone derivative	26.494	-	-	0.86
Benzophenone	C_13_H_10_O	Ketone	26.938	-	2.94	-
			26.918	-	-	2.70
1,3,5,7,9-Pentaethylbicyclo[5.3.1]pentasiloxane	C_10_H_28_O_6_Si_5_	Organosilicon compound	27.604	-	-	0.98
N,O-di(trimethylsilyl)-2-Aminophenol	C_12_H_23_NOSi_2_	Phenolic compound	28.189	4.99	-	-
3-methyl-nonadecane	C_20_H_42_	Alkane	28.270	-	-	2.61
Etymemazine	C_20_H_26_N_2_S	Amine	28.835	-	-	0.48
Octadecane	C_18_H_38_	Alkane	30.409	7.73	6.89	5.21
3-amino-3-oxopropanoic acid	C_3_H_5_NO_3_	Carboxyllic acid	31.902	-	-	1.14
1,2-Benzenedicarboxylic acid, butyl octyl ester-	C_20_H_30_O_4_	Carboxylic acid	33.819	5.50	1.34	2.78
Butyl nonyl ester phthalic acid	C_21_H_32_O_4_	Ester	33.859	-	2.88	-
Nonadecane	C_19_H_40_	Alkane	34.404	3.63	-	-
2,3-dihydroxypropyl 2-[(7-chloro-4-quinolinyl)amino]benzoate	C_19_H_17_ClN_2_O_4_	Ester	38.036	1.47	-	-
O-decylhydroxylamine	C_10_H_23_NO	Amine	34.444	-	-	2.33
			34.445	-	2.81	-
Hexadecanamide	C_16_H_33_NO	Amide	37.794	-	-	2.20
Heptacosane	C_27_H_56_	Alkane	38.077	-	1.68	-
Silicic acid, diethyl bis(trimethylsilyl) ester	C_10_H_28_O_4_Si_3_	silyl ester	42.153	-	-	1.74
Alkane’s identified (%)				71.5	69.2	37.5
Amines identified (%)				4.99	4.45	3.56
Carboxylic acids identified (%)				-	1.34	14.4
Organo-silicon compounds identified (%)				-	-	1.99
Esters identified (%)				3.23	2.88	8.39
Ketones identified (%)				-	-	3.56
Percentage of identified compounds (%)				85.2	80.8	73.3

(−): not applicable.

A total of sixty-seven (67) bioactive compounds were identified in the ethyl acetate extracts and hexane sample extracts across all the herbal concoction extracts analyzed using the GC-MS (Tables 2, 3). Samples extracted by ethyl acetate had forty (40) identified components while the samples extracted by hexane had twenty-seven (27) identified components. The overall percentage abundance of all the bioactive compounds identified in the hexane extracts of sample A, sample B and sample C are 97.5%, 96.8% and 83.7% respectively (Table 2). The overall percentage abundance of chemical constituents identified in the ethyl acetate extracts of samples A, B and C are 85.2%, 80.8% and 73.3% respectively. The prevalent compound in the hexane extract of sample C is octane (33.2%). The most abundant compound in the hexane extracts of the herbal concoction samples A and Sample B is 3-methylheptane at 29.1% and 30.4% respectively (Table 2). Hexadecane, tetradecane and 5-aminovaleric acid are the major constituents identified in the ethyl acetate extracts of samples A, B and C at 15.2%, 14.3% and 10.3% respectively (Table 3). Tetradecane which is one of the major constituents identified in ethyl acetate extracts has shown to exhibit diuretic, antibacterial, anti-tuberculosis and antifungal properties (Ozdemir et al., 2004; Girija et al., 2014). 5-Aminovaleric acid which is another compound identified in ethyl acetate extracts has been documented to show a significant suppression of development of seizure (epileptogenesis) in rats (Dhaher et al., 2014). Hexadecane was found in both hexane and ethyl acetate extracts, and it has antioxidant and antimicrobial activity (Yogeswari et al., 2012). *P*-xylene which is one of the compounds extracted by hexane, have antibacterial, antioxidant and antifungal properties (Aminsobhani et al., 2022; Akpuaka et al., 2013). Chemical constituents of ester, ketone and alcohol classification were identified with varying percentage abundance in the studied samples (Tables 2, 3). This may suggest that compounds of this chemical nature are widely spread in the herbal concoctions as they were identified in some herbal concoctions in Lesotho (Khoabane et al., 2023). 1, 2-Benzenedicarboxylic acid, dibutyl ester which is documented to exhibit indirect additive property (Waluyo and Wahyuni, 2021), is present in all the ethyl acetate extracts (Table 3). This compound was also identified in an anti-diabetic polyherbal mixture at 1.36% (Sigh et al., 2023). The notable biological activities of some of the chemical constituents present in the sample extracts studied through GC-MS profiling suggest that these herbal concoction samples might have pharmacological properties that are of therapeutic benefit.

Trace elements concentrations of three herbal concoction samples with their standard deviation values are displayed in Table 4. Ca was detected in high concentrations among the trace metals studied, with concentration range between 4.34 × 10^−1^ mg/kg and 2.36 × 10^2^ mg/kg. Trace element detected in low concentration across the samples is Mn with concentration range between 1.11 × 10^−2^ and 1.49 × 10^−2^ mg/kg (Table 4). Effiong and Udo (2010) and Kolasani et al. (2011), highlighted that medicinal plants that contain some prevailing Fe, Ca and K display excellent therapeutic action of the medicine. Trace elements which include Na and K were found in traditional herbal concoctions studied in South Africa (Matotoka and Masoko, 2017). Izah et al. (2022) reported varying elemental concentrations range in liquid herbal formulations from 0.565 to 6.94 mg/kg, 1.75–19.4 mg/kg and 0.00150–0.266 mg/kg for Mn, Fe and Co respectively. Trace elements like Mg, Na, K and Ca provides essential minerals that support an array of physiological functions in the body (Prashanth et al., 2015; Islam et al., 2023). Although trace elements are important for the overall wellbeing of humans, elevated levels have been reported to cause different health disorders (Theophanides and Anastassopoulou, 2002; Uriu-Adams and Keen, 2005). High Mn concentrations were found in patient’s urine admitted in a hospital who took traditional remedies linked with mobility and mortality (Steenkamp et al., 2000). Na and K which were detected in all the studied samples as shown in Table 4, plays a key role in acid-base balance in extracellular and intracellular fluids (Soetan et al., 2010). Ca is documented as a key component in strong bone, muscle, teeth formation and control of pre-menstrual syndromes (Noguer et al., 2001). Mg is an essential trace element for the proper growth of the body and plays a pivotal role in activities involving enzymatic control (Wallinga et al., 2000).

**TABLE 4:** Trace and heavy metal concentrations of sample A, B and C herbal concoctions.

Measured parameter	Sample A (mg/kg)	Sample B (mg/kg)	Sample C (mg/kg)
Cr	4.77 × 10^−3^ ± 7.55 × 10^−4^	3.74 × 10^−3^ ± 3.63 × 10^−4^	2.36 × 10^−3^ ± 5.83 × 10^−4^
Cu	5.61 × 10^−2^ ± 6.96 × 10^−3^	4.05 × 10^−1^ ± 8.98 × 10^−3^	1.93 × 10^−2^ ± 2.41 × 10^−3^
Pb	1.96 × 10^−2^ ± 1.47 × 10^−3^	2.34 × 10^−2^ ± 1.43 × 10^−3^	1.09 × 10^−2^ ± 5.30 × 10^−4^
Zn	6.63 × 10^−2^ ± 1.59 × 10^−3^	2.43 × 101 ± 3.63 × 10^−3^	7.17 × 10^−2^ ± 2.04 × 10^−3^
As	3.92 × 10^−3^ ± 1.55 × 10^−3^	ND	ND
Cd	2.74 × 10^−3^ ± 9.88 × 10^−4^	ND	1.52 × 10^−4^ ± 9.10 × 10^−5^
Ca	2.36 × 102 ± 3.81	1.46 × 102 ± 9.34 × 10^−1^	4.34 × 10^−1^ ± 1.14 × 10^−2^
K	2.26 × 101 ± 1.46 × 10^−1^	1.31 × 101 ± 1.04 × 10^−1^	1.13 ± 2.08 × 10^−2^
Mg	2.79 × 101 ± 8.93 × 10^−2^	2.07 × 101 ± 1.52 × 10^−2^	1.31 × 10^−1^ ± 3.00 × 10^−2^
Na	5.07 × 101 ± 3.13 × 10^−1^	3.36 × 101 ± 2.28 × 10^−1^	3.08 × 10^−1^ ± 1.10 × 10^−2^
Mn	1.49 × 10^−2^ ± 1.19 × 10^−3^	1.11 × 10^−2^ ± 3.75 × 10^−4^	1.16 × 10^−2^ ± 1.18 × 10^−4^

Elemental concentrations values are presented as mean of triplicates measurements ±standard deviation (SD). *n* = 3. ND, represent non detectable concentration.

Heavy metal concentrations of three herbal concoction samples with their respective standard deviation are displayed on Table 4. Sample B had non-detectable concentration levels of As and Cd. Sample C had non-detectable concentration level of As (Table 4). The non-detectable concentrations of As and Cd in the samples could be associated to the soil composition from which the plant species that make up the concoctions were harvested. Heavy metals concentrations ranged between 1.00 × 10^−4^ mg/kg and 2.43 × 10^−1^ mg/kg across the samples A, B and C (Table 4). The acceptable elemental concentration limits for Cr, Mn, Cu, Zn, Pb, As and Cd according to FAO/WHO are 0.02 mg/kg, 2 mg/kg, 3 mg/kg, 27.4 mg/kg, 10 mg/kg, 0.3 mg/kg and 0.2 mg/kg respectively (World Health Organization, 2007; Sadee, 2025). Saper et al. (2004) studied seventy (70) Ayurvedic herbal products which raised a significant safety concern of heavy metals present in traditional herbal preparations. It was discovered that 20.7% of the ayurvedic herbal products studied contained elevated concentration levels of As, Pb and Hg which were above the WHO permissible limits and also exceeded the daily intake thresholds set by United States environmental protection agency (Slope Factors (SF) for Carcinogens from US EPA, 2007) (Saper et al., 2004). Another study on herbal mixtures, reported elevated Pb and Cd concentrations on herbal mixtures above the permissible threshold set by WHO (Mousavi et al., 2014). Metal contamination in plants is linked to the uptake from polluted water and soil (Kacholi and Sahu, 2018). Contamination can also be caused by metal leaching from traditional processing tools and containers (Street, 2012). Moreover, it is also linked to the lack of standardization during preparation of the herbal formulations, poor handling and contamination during storage (Abou-Arab and Abou Donia, 2000; Saper et al., 2004). Abou-Arab and Abou Donia, (2000) found high Pb, Cu and Fe concentration levels in medicinal herbs after processing. In summary, all the elemental concentrations of sample A, B and C herbal concoctions are below the FAO/WHO standard permissible concentration levels. The non-detectable concentration levels of As and Cd suggest that the investigated samples may be safe for consumption as these heavy metals are toxic even in trace amounts (Tchounwou et al., 2004; Matta and Gjyli, 2016).

Table 5 shows SD, CI and CV results of trace concentrations in Samples A, B and C herbal concoctions. The CV values smaller than 20% are generally acceptable for bioanalytical methods depending on the nature of the matrix and the analyte concentration levels (US FDA, 2018; ICH Guideline, 2005). The studied samples standard deviation ranged between 1.18 × 10^−4^ and 3.81 with CV values range 6.46 × 10^−2^% and 7.99% (Table 5). T-test results, confidence intervals and relative variability results of heavy metals concentrations in sample A, sample B and sample C are displayed in Table 6. High (CV >20%) in Cd and As on sample A as shown in Table 7, may indicate high variability and low precision of the mean estimates of the true population mean which maybe be attributed to the non-standardized preparation techniques of the herbal mixtures, different plant species that make up the concoctions and soil composition (Anwar et al., 2024). The studied samples concentrations of Cd, Cr and Pb across the samples are statistically different (*p* < 0.05) from the WHO permissible values according to t-test as shown in Table 6 and this concords to Anwar et al. (2024) findings. Narrow CI values of Pb, Cd and Cr in sample C may suggest that the metal concentration is precisely estimated from the true population mean and may likely fall within the evaluated range (Table 6). One study on herbal mixtures, reported 14% of the ayurvedic herbal products with 95% confidence intervals; 11% lower limit and 31% upper limit, contains As, Pb and Hg (Saper et al., 2004). Earlier studies on traditional medicine and mixtures have shown that herbal mixtures and materials accumulate different metals through traditional preparation of these formulations, leaching due to using galvanized or aluminum pots during decoction and uptake from polluted environments (Saper et al., 2004; Street, 2012). The consumption of these herbal concoctions without thorough quality and safety checks present a silent and a potential public health risk. Therefore, there is need to perform comprehensive chemical analysis and safety profile of traditional herbal mixtures.

**TABLE 5:** Confidence intervals, standard deviation and coefficient of variation of trace elements concentrations in Samples A, B and C herbal concoctions.

Sample name	Elements	Elemental concentrations (mg/kg)	Standard deviation (SD)	95% confidence interval (CI) (mg/kg)	Coefficient of variation (CV %)
Sample A	Mn	1.49 × 10^−2^	1.19 × 10^−3^	1.19 × 10^−2^–1.79 × 10^−2^	7.99
	Na	5.07 × 101	3.13 × 10^−1^	4.99 × 10^−1^–5.14 × 101	6.17 × 10^−1^
	Mg	2.79 × 101	8.93 × 10^−2^	2.77 × 101–2.81 × 101	3.20 × 10^−1^
	K	2.26 × 101	1.46 × 10^−1^	2.25 × 101–2.26 × 101	6.46 × 10^−2^
	Ca	2.36 × 102	3.81	2.26 × 102–2.45 × 102	1.61
Sample B	Mn	1.11 × 10^−2^	3.75 × 10^−4^	1.02 × 10^−2^–1.20 × 10^−2^	3.38
	Na	3.36 × 101	2.28 × 10^−1^	3.28 × 10^−1^–33.9 × 10^−1^	6.83 × 10^−1^
	Mg	2.07 × 101	1.52 × 10^−2^	2.06 × 101–2.07 × 101	7.34 × 10^−2^
	K	1.31 × 101	1.04 × 10^−1^	1.28 × 10^−1^–1.34 × 10^−1^	7.94 × 10^−1^
	Ca	1.46 × 102	9.34 × 10^−1^	1.43 × 10^−2^–1.48 × 102	6.39 × 10^−1^
Sample C	Mn	1.16 × 10^−2^	1.18 × 10^−4^	1.13 × 10^−2^–1.19 × 10^−2^	1.02
	Na	3.08 × 10^−1^	1.10 × 10^−2^	2.8 × 10^−1^–3.35 × 10^−1^	3.57
	Mg	1.31 × 10^−1^	3.00 × 10^−2^	5.65 × 10^−2^–2.06 × 10^−1^	2.29 × 101
	K	1.13	2.08 × 10^−2^	1.07–1.18	1.84
	Ca	4.34 × 10^−1^	1.14 × 10^−2^	4.06 × 10^−1^–4.62 × 10^−1^	2.63

**TABLE 6:** T-test results, confidence intervals and relative variability of heavy concentrations in Samples A, B and C herbal concoctions.

Sample name	Elements	Elemental concentration (mg/kg)	Standard deviation (SD)	95% confidence interval CI (mg/kg)	Coefficient of variation (CV %)	WHO permissible concentration limits (mg/kg)	T-test	Interpretation
Sample A	As	3.92 × 10^−3^	1.55 × 10^−3^	6.96 × 10^−5^–7.78 × 103	3.95 × 101	3.0 × 10^−1^	P < 0.05	Significant
	Cd	2.74 × 10^−3^	9.88 × 10^−4^	2.85 × 10^−4^–5.19 × 10^−3^	3.61 × 101	3.0 × 10^−1^	P < 0.05	Significant
	Cr	4.77 × 10^−3^	7.55 × 10^−4^	2.89 × 10-3- 6.65 × 10^−3^	1.58 × 101	0.2 × 101	P < 0.05	Significant
	Cu	5.61 × 10^−2^	6.96 × 10^−3^	3.38 × 10^−2^–7.33 × 10^−2^	1.24 × 101	7.33 × 101	P < 0.05	Significant
	Pb	1.96 × 10^−2^	1.47 × 10^−3^	1.59 × 10^−2^–3.55 × 10^−2^	7.50	1.0 × 101	P < 0.05	Significant
	Zn	6.63 × 10^−2^	1.59 × 10^−3^	6.24 × 10^−2^–7.02 × 10^−2^	2.39	2.74 × 101	P < 0.05	Significant
Sample B	Cr	3.74 × 10^−3^	3.63 × 10^−4^	2.83 × 10^−3^–4.64 × 10^−3^	9.71	0.2 × 101	P < 0.05	Significant
	Cu	4.05 × 10^−1^	8.98 × 10^−3^	3.83 × 10^−1^–4.27 × 10^−1^	2.22	7.33 × 101	P < 0.05	Significant
	Pb	2.34 × 10^−2^	1.43 × 10^−3^	1.98 × 10^−2^–2.69 × 10^−2^	6.1	1.0 × 101	P < 0.05	Significant
	Zn	2.43 × 101	3.63 × 10^−3^	2.42 × 101–2.43 × 101	1.4 × 10^−3^	2.74 × 101	P < 0.05	Significant
Sample C	Cd	1.52 × 10^−4^	9.10 × 10^−5^	1.29 × 10^−3^–1.75 × 10^−3^	5.99	3.0 × 10^−1^	P < 0.05	Significant
	Cr	2.36 × 10^−3^	5.83 × 10^−4^	9.11 × 10^−4^–3.81 × 10^−3^	2.47 × 10^−3^	0.2 × 101	P < 0.05	Significant
	Cu	1.93 × 10^−2^	2.41 × 10^−3^	1.33 × 10^−2^–2.53 × 10^−2^	1.25 × 101	7.33 × 101	P < 0.05	Significant
	Pb	1.09 × 10^−2^	5.30 × 10^−4^	9.58 × 10^−3^–1.22 × 10^−2^	4.86	1.0 × 101	P < 0.05	Significant
	Zn	7.17 × 10^−2^	2.04 × 10^−3^	6.66 × 10^−2^–7.68 × 10^−2^	2.85	2.74 × 101	P < 0.05	Significant

**TABLE 7:** Monte Carlo probabilistic estimates of EDI, THQ, HI and CR for heavy metals in Samples A, B and C herbal concoctions.

Sample names	Metal	RfD	P50 EDI	P95 EDI	THQ (P50)	THQ (P95)	CSF	CR (P50)	CR (P95)
Sample A	As	3.00 × 10^−3^	4.48 × 10^−7^	7.45 × 10^−7^	1.49 × 10^−4^	2.48 × 10^−4^	1.5	6.72 × 10^−7^	1.12 × 10^−6^
	Cd	1.00 × 10^−3^	3.13 × 10^−7^	5.01 × 10^−7^	3.14 × 10^−4^	5.01 × 10^−4^	-	-	-
	Cr	2.00 × 10^−2^	5.45 × 10^−7^	6.86 × 10^−7^	2.72 × 10^−5^	3.43 × 10^−5^	0.5	2.73 × 10^−7^	3.43 × 10^−7^
	Cu	1.00 × 10^−3^	6.41 × 10^−6^	7.73 × 10^−6^	6.41 × 10^−3^	7.73 × 10^−3^	-	-	-
	Pb	4.00 × 10^−3^	2.24 × 10^−6^	2.51 × 10^−6^	5.60 × 10^−4^	6.27 × 10^−4^	8.5 × 10^−3^	1.90 × 10^−8^	2.13 × 10^−7^
	Zn	3.0 × 10^−1^	7.58 × 10^−6^	7.88 × 10^−6^	2.53 × 10^−5^	2.63 × 10^−5^	-	-	-
	Mn	1.40 × 10^−1^	1.70 × 10^−6^	1.93 × 10^−6^	1.22 × 10^−5^	1.38 × 10^−5^	-	-	-
HI	-	-	-	-	7.49 × 10^−3^	9.18 × 10^−3^	-	-	-
Sample B	Cr	2.00 × 10^−2^	4.28 × 10^−7^	4.94 × 10^−7^	2.14 × 10^−5^	2.47 × 10^−5^	0.5	2.14 × 10^−7^	2.47 × 10^−7^
	Cu	1.00 × 10^−3^	4.63 × 10^−5^	4.80 × 10^−5^	4.63 × 10^−2^	4.80 × 10^−2^	-	-	-
	Pb	4.00 × 10^−3^	2.68 × 10^−6^	2.94 × 10^−6^	6.69 × 10^−4^	7.36 × 10^−4^	8.5 × 10^−3^	2.27 × 10^−8^	2.50 × 10^−8^
	Zn	3.0 × 10^−1^	2.78 × 10^−3^	2.80 × 10^−3^	9.26 × 10^−4^	9.26 × 10^−4^	-	-	-
	Mn	1.40 × 10^−1^	1.70 × 10^−6^	1.93 × 10^−6^	1.22 × 10^−5^	1.38 × 10^−5^	-	-	-
HI	-	-	-	-	4.79 × 10^−2^	4.97 × 10^−2^	-	-	-
Sample C	Cd	1.00 × 10^−3^	1.04 × 10^−8^	1.32 × 10^−8^	1.04 × 10^−5^	1.32 × 10^−5^	-	-	-
	Cr	2.00 × 10^−2^	6.67 × 10^−8^	1.11 × 10^−7^	3.33 × 10^−6^	5.56 × 10^−5^	0.5	1.35 × 10^−7^	5.56 × 10^−8^
	Cu	1.00 × 10^−3^	2.75 × 10^−7^	3.37 × 10^−7^	9.18 × 10^−7^	1.12 × 10^−6^	-	-	-
	Pb	4.00 × 10^−3^	6.07 × 10^−8^	7.14 × 10^−8^	1.52 × 10^−5^	1.79 × 10^−5^	8.5 × 10^−3^	1.06 × 10^−8^	6.07 × 10^−10^
	Zn	3.0 × 10^−1^	2.33 × 10^−7^	2.58 × 10^−7^	7.76 × 10^−7^	8.60 × 10^−7^	-	-	-
	Mn	1.40 × 10^−1^	1.33 × 10^−6^	1.45 × 10^−6^	9.46 × 10^−6^	1.04 × 10^−5^	-	-	-
HI	-	-	-	-	4.01 × 10^−5^	9.91 × 10^−5^	-	-	-

EDI, Estimated daily intake (mg kg^−1^, day^−1^); RfD, Oral reference dose (mg kg^−1^, day^−1^); THQ, target hazard quotient; CSF, Cancer slope factor (mg/kg/day)^−1^; CR, cancer risk; HI, hazard index; P50, 50th percentile; P95, 95th percentile; -, not applicable.

Table 7 presents the results of Monte Carlo simulations estimating the EDI, THQ, HI and CR probabilistic health risks linked to ingestion of the heavy metals in the studied herbal concoctions, shown at the 50th percentile (P50) and 95th percentiles (P95). The P50 EDI and P95 EDI values of As, Cd, Cr, Cu, Pb and Mn were all in the range of 10^−8^ to 10^−6^, slightly lower than the oral reference dose (RfD) (Table 7). This indicates that the daily intake of these herbal concoctions poses no short term to mid-term adverse effects health effects to the population consuming them. Cu in Sample B exhibited the highest EDI in P50 and P95 (2.78 × 10^−3^ mg kg^−1^ day^−1^), whereas As, Pb, Mn and Cr displayed a relatively lower exposures. These lower evaluated EDI values are in congruence with previous studies on exposure assessment conducted in herbal concoctions teas and algal formulations (Durodola et al., 2019; EFSA, 2012). The ascending order from Monte Carlo probabilistic estimates of THQ values (at P50 and P95) for both sample A and sample B herbal concoctions are; Cu > Pb > Cd > As > Cr > Zn > Mn, Cu > Zn > Pb > Cr > Mn and the ascending order for sample C THQ values (at P50 and P95) are Pb > Cd > Mn > Cr > Cu > Zn, Cr > Pb > Cd > Mn > Cu > Zn respectively. Cu demonstrated the highest THQ values in both Sample A (6.41 × 10^−3^ at P50 and 7.73 × 10^−3^ at P95) and Sample B (4.63 × 10^−2^ at P50 and 4.80 × 10^−2^ at P95. The THQ values in both P50 and P95 of each heavy metal across all the samples is less than one (Table 7). Therefore, there is no health risk to the population consuming the herbal concoctions and protective measures and interventions are not urgently needed to protect the exposed population (Wang et al., 2005; Ekhator et al., 2017; Monang et al., 2016). The combined effects of heavy metals present in the herbal concoctions is unlikely to pose health risk in the long term to the adult population consuming them, since their HI is less than 1 (Table 7). The CR values for P50 and P95 in As, Cr and Pb in all the studied samples were below 10^−4^ (Table 7). CR values more than 1 × 10^−4^, suggest a potential risk of cancer and the values in the range between 1 × 10^−6^ and 1 × 10^−4^ are acceptable. The CR values below 1 × 10^−6^ are deemed negligible (Alawadhi et al., 2024; Saah et al., 2024). Sample A exhibited the highest CR value of As (1.12 × 10^−6^ at P95), followed by Pb (2.13 × 10^−7^ at P95) and Cr (1.35 × 10^−7^ at P50) in sample C (Table 7). Notably, these CR values are within the acceptable CR thresholds, suggesting no long term carcinogenic risk to the exposed population consuming the herbal concoctions. Similar health risk levels are documented in studies on heavy metal exposure through inhalation and ingestion pathways (Li et al., 2014; Sadee, 2025; Saeedi et al., 2012). Herbal concoction teas in Nigeria show no potential health risk as shown by EDI, THQ and CR evaluations and Co (54.18%) was identified as the major cause of concern from the relative risk (RR) results (Durodola et al., 2019). Another study on traditional herbal medicines reported HI values > 1 for As and about 50% of the herbal products had Pb and Cd HI values above the threshold, posing a potential health threat to the exposed population (Anwar et al., 2014). The Monte Carlo based non-carcinogenic and CR analysis suggest that these herbal concoctions may be safe for consumption, even though As concentration levels may require some close scrutiny as chronic exposure of As even at low concentration levels can cause cancer (Chakraborti et al., 2016). However, detailed toxicity studies are needed to further characterize the health risks of the herbal concoctions for safety to be confirmed and ascertained. These study findings bridge the gap between traditional knowledge and scientific validation of herbal concoctions and may inform a regulatory review, frameworks and foundation for future studies on the safety and efficacy of the herbal concoctions.

## 4 Conclusion

The FT-IR spectra of the three samples revealed the presence of functional groups such as-alkene, carbonyl, alcohols, alkanes, hydroxyl and aromatic groups, which are characteristic of some phenolic, aromatic compounds which were identified through GC-MS analysis. A total of sixty-seven (67) bioactive compounds were identified by GC-MS analysis in the ethyl acetate and hexane extracts of the samples A, B and C. 2,3-dihydroxypropyl-2-[(7-chloro-4-quinolinyl)amino]benzoate, 3-methylheneicosane and Bicyclo[2.1.1]hexan-2-ol, 2-ethenyl are the least abundant compounds found in samples A, B and C ethyl acetate extracts at 1.47%, 0.78% and 0.15% respectively. The least abundant chemical constituents identified across hexane extracts are 1-Monolinoleoylglycerol trimethylsilysl ether (1.58%), 6-methyl-octadecane (1.56%) and dodecane (0.35%). Heavy metal concentration across all the samples ranged between 1.00 × 10^−4^ mg/kg and 2.43 × 10^1^ mg/kg and were below the acceptable metal concentration levels specified by WHO/FAO. Trace elements, Ca, Mg, K and Mn were all detected across the samples. Arsenic was non-detectable in sample B and C. Cu > Pb > Cd > As > Cr > Zn > Mn, Cu > Zn > Pb > Cr > Mn is the ascending order of both THQ values at 50th percentile and 95th percentile for sample A and sample B herbal concoctions and Pb > Cd > Mn > Cr > Cu > Zn, Cr > Pb > Cd > Mn > Cu > Zn is the ascending order for sample C THQ values at 50th percentile and 95th percentile respectively. The THQ and HI of all the studied metals is below 1 in all the samples and this indicates that there is no potential long term health risk to the exposed population. The carcinogenic risk exhibited by all the herbal concoctions is below the set tolerable limit. Although the health risk assessment showed that the concentrations of the investigated heavy metals are unlikely to pose potential health risk to the exposed population at the current concentration levels, moderate consumption of the herbal concoctions is advised to prevent bioaccumulation of the metals. The study findings are important in that they provide valuable information on the chemical makeup; bioactive compounds present, metal composition (Trace and heavy metals) and health risk assessment of heavy metals identified in the herbal concoctions. This is first study in Botswana to apply different multidisciplinary analytical approach to thoroughly profile chemically, multi-component medicinal plant remedies (herbal concoctions). Additionally, the findings also provide important health risk assessment information for public health agencies, the pharmacovigilance systems and the exposed population on the safety of the herbal formulations. Further research *in vitro* and *in vivo* acute and chronic toxicity studies, bioactivity guided fractionation and assays to validate the activity of the identified bioactive constituents, compound isolation and purification, evaluating the nutritional content of the concoction extracts, increasing the sample size and geographical area are recommended. The limited sample size (n = 3) and the baseline FT-IR-corrected replicates and spectral enhancement were not done as the samples were safely discarded post analysis by following the laboratory and biosafety regulations are the key limitations of the study.

## Data Availability

The raw data supporting the conclusions of this article will be made available by the authors, without undue reservation.
